# Application of neutron imaging in observing various states of matter inside lithium batteries

**DOI:** 10.1093/nsr/nwad238

**Published:** 2023-09-15

**Authors:** Lei Gao, Songbai Han, Haijin Ni, Jinlong Zhu, Liping Wang, Song Gao, Yonggang Wang, Dubin Huang, Yusheng Zhao, Ruqiang Zou

**Affiliations:** School of Materials Science and Engineering, Peking University, Beijing 100871, China; Academy for Advanced Interdisciplinary Studies, Southern University of Science and Technology, Shenzhen 518055, China; Department of Physics, Southern University of Science and Technology, Shenzhen 518055, China; Department of Physics, Southern University of Science and Technology, Shenzhen 518055, China; Academy for Advanced Interdisciplinary Studies, Southern University of Science and Technology, Shenzhen 518055, China; School of Materials Science and Engineering, Peking University, Beijing 100871, China; School of Materials Science and Engineering, Peking University, Beijing 100871, China; School of Materials Science and Engineering, Peking University, Beijing 100871, China; Eastern Institute for Advanced Study, Ningbo 315201, China; School of Materials Science and Engineering, Peking University, Beijing 100871, China

**Keywords:** lithium battery, neutron imaging, electrodes, electrolytes

## Abstract

Lithium batteries have been essential technologies and become an integral part of our daily lives, powering a range of devices from phones to electric vehicles. To fully understand and optimize the performance of lithium batteries, it is necessary to investigate their internal states and processes through various characterization methods. Neutron imaging has been an indispensable complementary characterization technique to X-ray imaging or electron microscopy because of the unique interaction principle between neutrons and matter. It provides particular insights into the various states of matter inside lithium batteries, including the Li^+^ concentration in solid electrodes, the Li plating/stripping behavior of Li-metal anodes, the Li^+^ diffusion in solid ionic conductors, the distribution of liquid electrolytes and the generation of gases. This review aims to highlight the capabilities and advantages of neutron imaging in characterizing lithium batteries, as well as its current state of application in this field. Additionally, we discuss the potential of neutron imaging to contribute to the ongoing development of advanced batteries through its ability to visualize internal evolution.

## Teaser

Neutron imaging is an indispensable technique for understanding batteries by providing unique insights into their internal states and processes, including lithium ion concentration, Li-metal anode behavior, electrolyte distribution, and gas generation.

## INTORDUCTION

Batteries have been extensively used in intelligent electronics, electric vehicles and smart grids, etc., due to their characteristics of high open-circuit voltage, large discharge capacity and long cycle life [1]. However, driven by the mushroom growth of global economy and technology, the energy density, cycle life and safety of current batteries are not satisfactory for large-capacity energy storage (i.e. renewable solar and wind energy sources) or the expansion of the vehicle electrification [2].

In general, the development of innovative materials, breakthrough in new-type batteries and the amelioration of assembly processes are considered effective ways to improve the performance of batteries. For instance, originating from transition metal ion redox reactions and oxygen anion redox reactions, Li-rich cathode materials have exhibited high reversible discharge capacity (>250 mAh·g^−1^) in lithium-ion batteries (LIBs) [3]. The use of stable lithium salts or adding flame retardant additives to nonaqueous electrolytes can effectively reduce the burning risk of LIBs [4]. All-solid-state battery technologies can provide promising solutions to safety issues and energy density limitations [5].

On the other hand, employing advanced characterization techniques to monitor the operation status of batteries and analyze functional materials also plays an important role in advancing battery technologies[6–8]. Over the last few decades, as the practical methods of characterization, imaging techniques are extensively used to observe the morphology of electrode materials, electrolytes and internal architecture of batteries [9–11]. For example, optical microscopy (OM) can directly observe the morphology evolution of the electrode layers and detect the macroscopical heterogeneity of electrochemical reactions in batteries, such as the nonuniform lithium plating/stripping during cycling or the growth of dendrites on the Li metal anode [12–14]. However, OM is limited to providing surface-level images and cannot capture the intricate behaviors of electrodes and electrolytes within the batteries. Atomic force microscopy (AFM), as a surface analysis technique, has advantages in studying the dynamic change process of the solid electrolyte interface (SEI) layer in LIBs, since it is suitable for acquiring high-resolution surface morphology in a variety of sample environments (such as liquid and temperature) [15–17]. But the limited scan speed and size of AFM constrain its broader application in lithium batteries. Electron microscopy (EM), particularly *in situ*, is a powerful imaging technique that is widely used to provide insight into electrode materials at the atomic resolution, and combine with other spectroscopy techniques to obtain elemental mapping, and chemical valence information [18–21]. However, the application of EM is limited by the demanding test environment (i.e. high vacuum), the short transmission depth of electrons (<10^−6^ m) and the damage of high-energy electron beams to battery materials [9,22–24]. Compared with the electron beam, X-ray causes less damage to the battery materials. Therefore, as a nondestructive characterization method, X-ray computed tomography (CT) technology has demonstrated its advantages in visualizing the internal structure, electrode deformation and active material particles of batteries [25–27]. With the exploration of numerous new battery materials and the rapid progress of battery technology, the demands on X-ray CT involving a wider range of material characterizations have expanded. It is not only necessary to obtain information about the solid phase such as the electrodes, separators and current collectors during the charge and discharge of batteries, but also to observe the evolution process of internal liquid and gas substances [28,29], including the consumption of liquid electrolyte and the gas production associated with the performance deterioration. However, X-rays exhibit insensitive to light elements, and the attenuation is not obvious when passing through low-density substances (i.e. liquid electrolytes and gas). Therefore, internal information about the batteries cannot be comprehensively displayed by X-ray CT.

**Figure 1.**
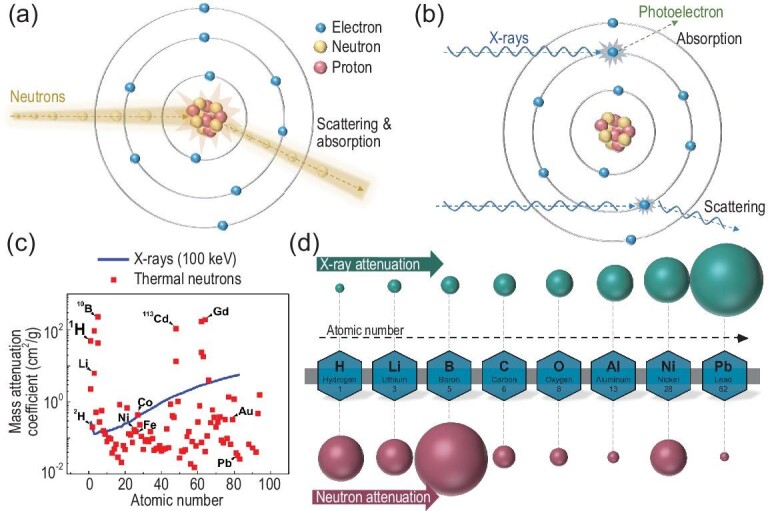
Different mechanisms of interaction between neutrons/X-rays and matter. (a) Interaction between neutrons and nuclei. (b) Interaction between X-rays and electrons. (c) Mass attenuation coefficients (MAC) for thermal neutrons and X-rays (100 keV). MAC almost monotonically depends on the atomic number for X-rays, while there is no dependency between MAC and the atomic number for neutron. Reprinted with permission from ref. [37]. Copyright 2008, Oxford Academic. (d) Comparison diagram of neutron and X-ray attenuation of different elements.

The limitations of X-ray imaging aforementioned are primarily attributed to the inherent mechanism of the interaction between X-rays and matter (Fig. 1(a) and (b)) [30–32]. When X-rays pass through materials, they mainly interact with electrons outside the nuclei of the atoms. Hence, the interaction cross section between X-rays and atom increases with atomic number (Fig. 1(c)) [33–37]. In contrast, uncharged neutrons do not interact with the electrons outside the nuclei, but exhibit a strong interaction with the nuclei. This results in the mass attenuation coefficient nonmonotonically changing with increasing atomic number. For instance, there are obvious differences in the interaction cross sections between neutrons and some adjacent elements, and isotopes can even be distinguished. Significant attenuation appears, especially when neutrons interact with specific light elements (e.g. lithium), while some heavy element metals (e.g. lead) can be easily penetrated by neutrons (Fig. 1(d)). Therefore, neutron imaging (NI), as an indispensable complement to X-ray imaging or electron microscopy, shows its significant advantages in characterizing batteries from the perspective of the interaction principle between rays and matter. Neutron detection for imaging typically involves a two-step process. Firstly, chargeless neutrons are captured by a potent neutron absorber, e.g. the lithium isotope ^6^Li or natural gadolinium. During this capture process, charged particles or X-rays are emitted. Subsequently, the secondary radiation travels a certain distance and interacts with a scintillating material, e.g. ZnS, generating visible light that can be detected by conventional position-sensitive detectors like CCD or CMOS cameras [33]. Typically, NI possesses visible contrast to the internal components (solid, liquid and gaseous phases) of batteries, because Li or H elements widely exist in electrodes, Li metal and electrolyte. In other words, in terms of function and applicable objects, NI can comprehensively observe various states of matter and phenomena within batteries, surpassing other imaging methods. Additionally, Supplementary Table S1 lists neutron scattering lengths and cross sections of some common elements and their isotopes in lithium batteries for quick reference [38].

In this review, we focus on the application of NI in observing different states of matter—classified by solid, liquid and gaseous phases—inside lithium batteries. Also, the current development of the NI technique and possible future directions for its application in advanced batteries are discussed.

## SOLID PHASE

In solid electrode materials, the lithium concentration typically fluctuates significantly during charge and discharge [39–42]. Lithium possesses a large attenuation coefficient for neutrons, which implies that there will be differences in imaging contrast, when neutrons pass through the solid electrode materials with varying lithium concentration gradients. This feature can, on the one hand, be used to quantitatively assess the lithiation/delithiation state of the electrode and the capacity of batteries [43–54]. On the other hand, nondestructive NI allows for the observation of inhomogeneity in lithium electrodeposits on the anode, volume change of Li metal and lithium dendrite growth can be observed *in situ* by nondestructive NI [55–60], which compensates for the deficiency of X-ray imaging in the analysis of Li metal. In addition, the NI technique can measure the tracer diffusion coefficient of lithium ion in electrode or electrolyte materials using diffusion couples with different isotope concentrations [61–65].

### Lithium distribution in electrodes

**Figure 2.**
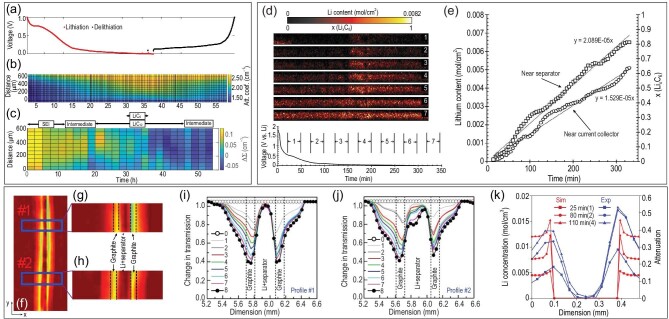
Application of NI in the analysis of lithiation/delithiation of a graphite electrode. (a) Voltage profile for the first lithiation/delithiation cycle of a Li/graphite cell; (b) attenuation coefficient map of a graphite electrode; (c) map of the relative attenuation change with 2-h increments. Reprinted with permission from ref [43]. (d) Lithium distributions in a graphite electrode during the first discharge presented by time-resolved NI, and the voltage response at a C/9 discharge rate; (e) lithium insertion measured with NI near the separator and current collector. Reprinted with permission from ref [45]. Copyright 2012, Elsevier. (f–h) The regions of interest (marked by blue rectangles, #1 and #2) for line profile analyses; (i and j) transmission profiles of #1 and #2 regions gradually decrease with discharging (from step 1 to step 8); (k) comparison of the calculated lithium concentration as a function of the electrode thickness (red curves) to the experimental line profiles of the attenuation (blue curves) at discharge times of 25, 80 and 110 min. Reprinted with permission from ref [51]. Copyright 2016, American Chemical Society.

The hexagonal layers in graphite are linked by van der Waals forces and $\pi-\pi$ delocalized orbital interactions, which allows reversible intercalation of Li ions along with the formation of lithiated graphite phases during electrochemical cycling of batteries [66]. Strobl *et al.* [43] demonstrated the lithium distribution and SEI formation in graphite electrodes based on the local beam attenuation of NI, throughout the lithiation/delithiation process of a Li/graphite cell (Fig. 2(a)– (c)). It was observed *in situ* that the attenuation coefficient of the whole graphite electrode gradually increased during discharge (Fig. 2(b)). Notably, the map of relative attenuation change indicated that the SEI formation possessed a slight gradient from the separator interface deeper in the graphite electrode, and slowed down at approximately 10 h (Fig. 2(c)). This result visually verified that the shoulder in the discharge curve (within 10 h) appeared at a voltage between 0.8–0.2 V (versus Li/Li^+^) can be related to the formation of SEI (Fig. 2(a)). Also, the intercalation of lithium in graphite can be quantified with NI using on-line calibration [45]. Owejan *et al.* [45] assumed that the attenuation change for graphite electrodes was only related to lithium insertion, and proposed a linear model to approximate the increase in transport resistance. The high-resolution NI suggested that a nonuniform lithium concentration distribution existed early during discharging at a C/9 rate, but relaxed to a more homogeneous distribution at low potentials (Fig. 2(d)). The difference in lithiation rates near the separator and current collector revealed that a through-plane transport resistance existed in the bulk of the graphite electrode (Fig. 2(e)). By analyzing the lithium content at the fully charged state, the irreversible capacity loss associated with residual lithium was also quantified. Zhou *et al.* [51] correlated the changes in NI contrast to the state of lithiation *in situ* in a pouch cell. As shown in Fig. 2(f)– (h), data analysis was performed at two different regions of interest (marked by blue rectangles #1 and #2). The peak of the transmission profile gradually decreases with discharging (from step 1 to step 8), indicating lower neutron transmission as a function of lithiation, which could reach a maximum at the end of the discharge (Fig. 2(i) and (j)). The change of transmission is distinct for each profile for the two graphite electrodes even at the same discharge step, implying the heterogeneous intercalation of lithium, and the profiles shift toward the bulk of the graphite may be related to the volume expansion caused by lithiation. Besides, the neutron attenuation or lithium concentration at different states of charge (SoCs) is qualitatively consistent with the results calculated based on an electrochemical transport model (Fig. 2(k)).

**Figure 3.**
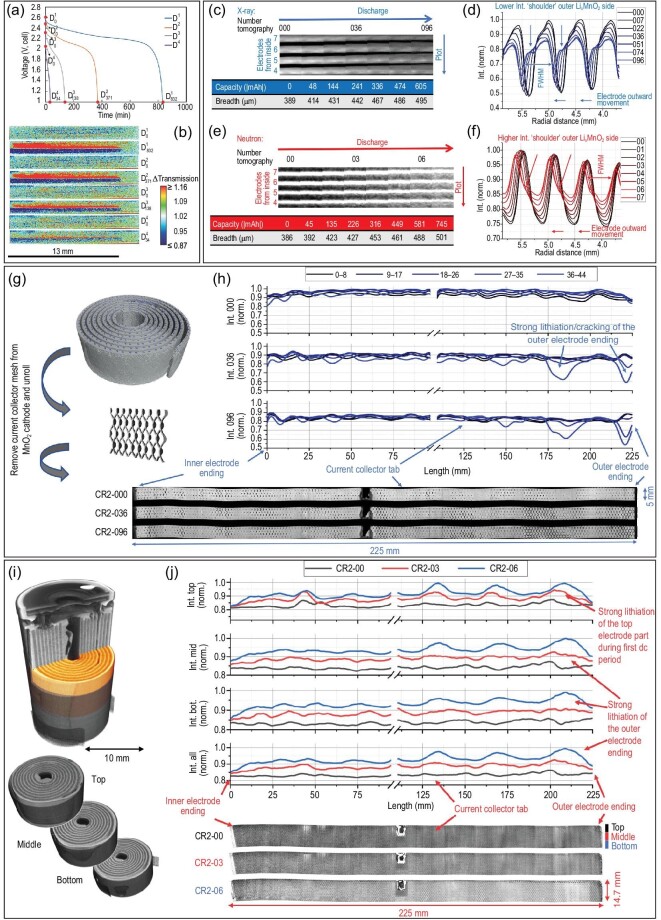
Application of NI in the analysis of lithiation/delithiation of a cathode electrode. (a) Discharge profiles at C/20 (blue), C/10 (orange), C/5 (grey) and C/2.5 (purple); the points labeled D$^i_x$ represent the *x*th minute in the *i*th discharge process; (b) the neutron imaging radiographs corresponding to the points noted in (a). Reprinted with permission from ref [52]. Copyright 2020, Royal Society of Chemistry. (c–f) Cutouts of virtual unrolled multilayer sections of the measured X-ray and neutron tomograms at different SoCs, and the thickness of the Li$\rm _x$MnO_2_ cathode electrode and the intensity plot change during discharge; (g and h) lithium distribution at various depths of the Li$\rm _x$MnO_2_ electrode during discharging, using unrolled X-ray tomography data of the upper part of a CR2 cell; (i and j) organization of electrode unrolling (top, middle, bottom) and analysis of the lithium distribution in the Li$\rm _x$MnO_2_ electrode during discharging using neutron tomography data. Reprinted with permission from ref [56]. Copyright 2020, Springer.

Moreover, NI embodies its superiority in monitoring the lithiation/delithiation of cathode electrodes during charge/discharge. Nie *et al.* [52] employed the NI technique to track Li^+^ intercalation/deintercalation in a full cell with thick sintered electrodes at different discharge rates. The neutron images revealed that Li^+^ transferred from the Li_4_Ti_5_O_12_ (LTO) anode to the LiCoO_2_ (LCO) cathode was highly dependent on the discharge rates, which was the visual evidence of the correlation between discharge capacity and current density. This provided visual evidence of the correlation between discharge capacity and current density (Fig. 3(a) and (b)). In addition, the calculation based on a numerical model indicated that the tortuosity had a significant effect on the discharge performance at a higher discharge rate, while it did not at a lower rate. Ziesche *et al.* [56] evaluated the lithium distribution in the MnO_2_ cathode of a commercial CR2 Li-ion primary cell using both X-ray and neutron CT. As shown in Fig. 3(c)– (f), both X-ray and neutron images revealed the volume expansion and electrode movement with the lithiation of Li$\rm _x$MnO_2_ at different discharge capacities. Through virtual electrode unrolling techniques processing of X-ray and neutron images, heterogeneous fluctuations of the sinusoidal-shaped intensity over the full length of the Li$\rm _x$MnO_2_ electrode were identified. This indicated higher lithium intercalation at the outer cell windings caused by a lower compression of the active material (Fig. 3(g)–(j)).

Indeed, whether for graphite anode or cathode materials, the application of NI to analyze the distribution of lithium in electrodes is primarily rooted in the fact that lithium exhibits a significant attenuation coefficient for neutrons. As neutrons traverse through electrode materials with varying gradients of lithium concentration, distinct disparities in imaging contrast arise. Moreover, this characteristic holds immense potential for investigating the lithiation/delithiation processes and evaluating the capacity performance of state-of-the-art electrode materials in forthcoming studies.

### Li-metal anode

**Figure 4.**
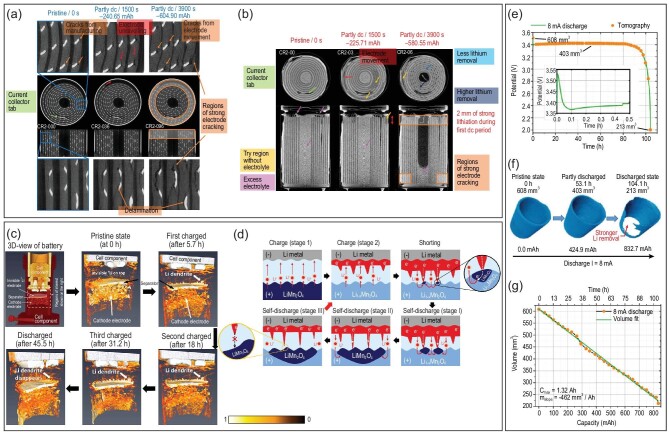
Application of NI in observing the dynamic evolution process of the Li-metal anode. (a) Three-dimensional (3D) reconstructed operando X-ray tomograms of a commercial Li/MnO_2_ primary battery; (b) 3D reconstructed *in situ* neutron tomograms of a commercial Li/MnO_2_ primary battery. Reprinted with permission from ref [56]. Copyright 2020, Springer. (c) 3D evolution of Li metal and lithium dendrite in a Li/LiMn_2_O_4_ battery at different stages of charging and discharging; (d) schematic illustrations of the competing mechanism between charge and short-induced self-discharge after battery shorting. Reprinted with permission from ref [57]. Copyright 2019, American Chemical Society. (e) Discharge curve of a Li/SOCl_2_ cell at 8 mA; (f) volume renderings of the Li-metal anode discharged at different SoCs with the pristine, half and fully discharged states; (g) the linear relationship between discharge capacity and Li-metal volume. Reprinted with permission from ref [59]. Copyright 2020, Institute of Physics.

Li metal possesses a high theoretical specific capacity (3860 mAh·g^−1^) and an ultralow electrochemical potential (−3.04 V versus the standard hydrogen electrode), thereby Li metal has been referred to as the ‘holy grail’ anode of lithium batteries to fulfill the need for energy density [67–69]. However, the challenges of lithium dendrite growth [70], SEI formation [71] and volume change [72] hinder the widespread application of Li metal in batteries. Hence, it is essential to *in situ* observe the dynamic evolution process of Li metal in batteries. Compared with X-ray imaging, neutron imaging exhibits its superiorities in observing Li metal. As Fig. 4(a) indicates, X-ray is a good probe for detecting mechanical degradation processes (e.g. cracking, delamination, unravelling) in the Li$\rm _x$MnO_2_ electrode, but it is difficult to distinguish the morphology of Li metal. By contrast, a neutron is favorable to detect the consumption of the Li-metal anode during discharge because of its remarkable attenuation coefficient to lithium (Fig. 4(b)) [56]. Song *et al.* [57] performed an operando experiment using neutron radiography (two dimensions) and static tomography (three dimensions) to investigate the dynamic evolution process of Li metal, when the Li/LiMn_2_O_4_ battery was shorted internally. Interestingly, the lithium dendrite on Li metal exhibited progressive growth at different stages of charging, while vanishing at the end of discharging (Fig. 4(c)). Accordingly, they inferred that the short-circuit dominated by the lithium dendrite would lead to a continuous drop in the charging curve, while the subsequent fluctuating charging profile was attributed to the competition mechanism between charge and self-discharge caused by internal shorting (Fig. 4(d)). Also, the Li-metal volume over the discharge time can be quantitatively calculated by neutron tomography. As presented in Fig. 4(e) and (f), the Li-metal anode showed distinct Li removal and its volume changed from 608 to 213 mm^3^, after a complete discharge of a primary Li/SOCl_2_ cell at 8 mA. Meanwhile, there was a linear relationship between the volume of Li metal and the discharge capacity (Fig. 4(g)), which meant that the potential battery capacity can be predicted by the volume of residual Li metal determined by neutron tomography [58,59]. The aforementioned studies highlight the advantages of NI in monitoring volume variations of Li metal and the growth of lithium dendrites, which indicates that NI is a powerful tool for analyzing the interface evolution mechanism of the Li-metal anode and quantitatively evaluating the SoC in Li-metal batteries.

### Diffusion coefficient

**Figure 5.**
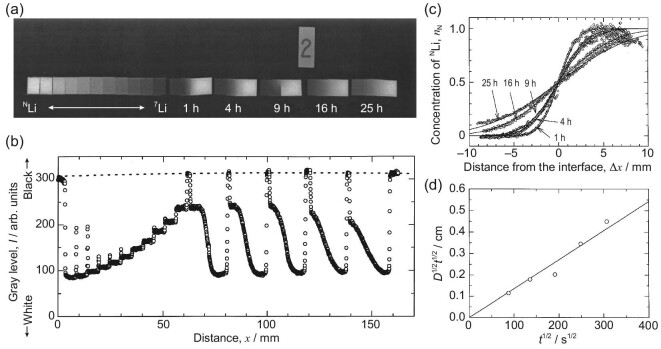
Application of NI in measuring the diffusion coefficient of lithium ions in sintered Li_1.33_Ti_1.67_O_4_. (a) Neutron image of the standard samples, annealed diffusion couples of Li_1.33_Ti_1.67_O_4_ and diffusion annealing, carried out at 900^○^C for 1, 4, 9, 16 and 25 h; (b) digitized gray level of the neutron image observed in (a); (c) the $\rm ^N$Li concentration curves and calculated diffusion plots by nonlinear least-squares fitting at 900^○^C for 1, 4, 9, 16 and 25 h; (d) relationship between *D*^1/2^*t*^1/2^ obtained by profile fitting and the square root of the actual annealing time. Reprinted with permission from ref [62]. Copyright 1999, Elsevier.

The attenuation coefficient for the neutron beam, unlike X-rays, is independent of the atomic number, which gives NI an advantage in identifying some adjacent elements and even isotopes [73,74]. In particular, there is a large difference in the neutron absorption cross section between ^6^Li and ^7^Li isotopes. For instance, when exposed to thermal neutrons with an energy of 25 meV, ^6^Li demonstrates a considerable absorption cross section of 940 b. In contrast, ^7^Li behaves nearly transparently with an absorption cross section of 0.0454 b [38]. Therefore, the neutron beam is significantly attenuated in materials containing ^6^Li and can easily pass through a ^7^Li atom, which allows for the visualization of the diffusion profile of lithium ions in electrode/electrolyte materials and the analysis of the tracer diffusion coefficient. Takai *et al.* [62] applied the NI technique to measure the tracer diffusion coefficient of lithium ions in sintered Li_1.33_Ti_1.67_O_4_. As presented in Fig. 5(a), the lithium isotope ratio of standard samples was varied every 1/10 step from $\rm ^N$Li to ^7^Li, and annealing was carried out for diffusion couples of Li_1.33_Ti_1.67_O_4_ at 900 ^○^C. By comparing it with the calibration curve based on the digitized gray level of the neutron image (Fig. 5(b)), the concentration profiles can be fitted by Fick’s equation at different annealing times (Fig. 5(c)), and then the diffusion coefficient *D* can be obtained through approximately linear fitting (Fig. 5(d)). Likewise, the NI technique can be applied to calculate the tracer diffusion coefficients of the lithium superionic conductor (LISICON). By analyzing the relationship between mobility and the diffusion coefficient, the difference in the diffusion coefficient of LISICON was considered to be due to the jump frequencies of lithium-ion interstitials rather than the migration path [65]. The application of NI in studying the behavior of lithium-ion diffusion is founded on the notable disparity in the neutron absorption cross section between the isotopes ^6^Li and ^7^Li. It not only enables the investigation of lithium-ion conduction in bulk materials, but also facilitates the exploration of lithium-ion diffusion at the interfaces between solid materials.

## LIQUID PHASE

The electrolyte is the essential component to ensure the transport of lithium ions between the cathode and anode during charging and discharging [75,76]. Generally, the liquid organic electrolyte contains Li and H elements, making it suitable for *in situ* analysis with NI due to the strong neutron beam attenuation [77]. The evolution of electrolyte mainly involves the soaking during the filling process [78,79], and the consumption during the operation of LIBs [80–82].

### Electrolyte filling process

**Figure 6.**
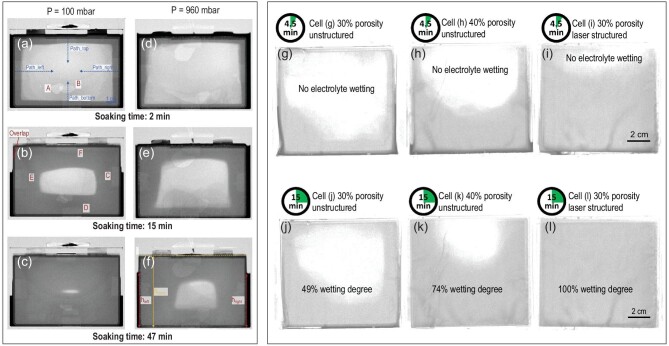
Application of NI in observing the electrolyte filling process of LIBs. (a–c) Normalized images of electrolyte soaking in hard case prismatic cells under ambient pressure after 2, 15 and 47 min; (d–f) normalized images of electrolyte soaking in hard case prismatic cells under vacuum after 2, 15 and 47  min. Reprinted with permission from ref [87]. Copyright 2018, Elsevier. (g and j) Wetting degree of a cell with unstructured electrodes of 30% porosity after 4.5 and 15 min; (h and k) wetting degree of a cell with unstructured electrodes of 40% porosity after 4.5 and 15 min; (i and l) wetting degree of a cell with laser structured electrodes of 30% porosity after 4.5 and 15 min. Reprinted with permission from ref [79]. Copyright 2019, Springer.

The process of filling electrolytes is time consuming and critical to the performance of LIBs [83,84]. After the liquid organic electrolyte solution is injected into the void space in a cell, wetting or soaking occurs, i.e. the electrolyte is absorbed into the electrode stack. The electrode structure and the setting conditions (e.g. pressure) during the filling process have a significant impact on the spreading speed and state of the liquid electrolyte [85–87]. Weydanz *et al.* [87] applied NI for the first time to visualize *in situ* the soaking process of electrolytes and study the effect of vacuum on the wetting speed in commercial hard case prismatic cells. As presented in Fig. 6(a) and (d), edges around the electrode stacks were wetted and appeared darker than other regions after 2 min of liquid electrolyte filling into the cells. As the electrode stacks absorbed the electrolyte, the wetting fronts advanced from edges at a similar speed towards the middle region, and the amount of excess electrolyte at the sides decreased. After 47 min of electrolyte soaking (Fig. 6(c)), almost all areas of the electrode stack were soaked by electrolyte at vacuum (100 mbar). In contrast, a large portion in the center of the electrode stack remained dry at ambient pressure (960 mbar; Fig. 6(f)). Habedank *et al.* [79] studied the influence of porosity and laser structuring of electrodes on the wetting behavior of pouch cells by NI. The conventional electrodes with 30% porosity (Fig. 6(g) and (j)) presented a slower soaking speed of electrolyte than the electrodes with 40% porosity (Fig. 6(h) and (k)), because the large pore volume in electrodes with 40% porosity was favorable for fluid dispersion with lower resistance. Besides, laser-structured electrodes exhibited a much faster soaking speed and were completely wetted in 15 min, even when the porosity of the electrodes was only 30% (Fig. 6(i) and (l)). From the perspective of *in situ* dynamic evolution of electrolyte dispersion, this study, with the assistance of NI, showed that laser structuring of electrodes may play an important role in reducing the time consumed and the production costs of LIBs. These *in situ* observations of the electrolyte filling process underscore the distinctive advantage of NI in analyzing the distribution of electrolyte within LIBs. This capability is crucial for effectively managing the soaking time of the electrolyte and reducing the production cost of LIBs.

### Electrolyte consumption

**Figure 7.**
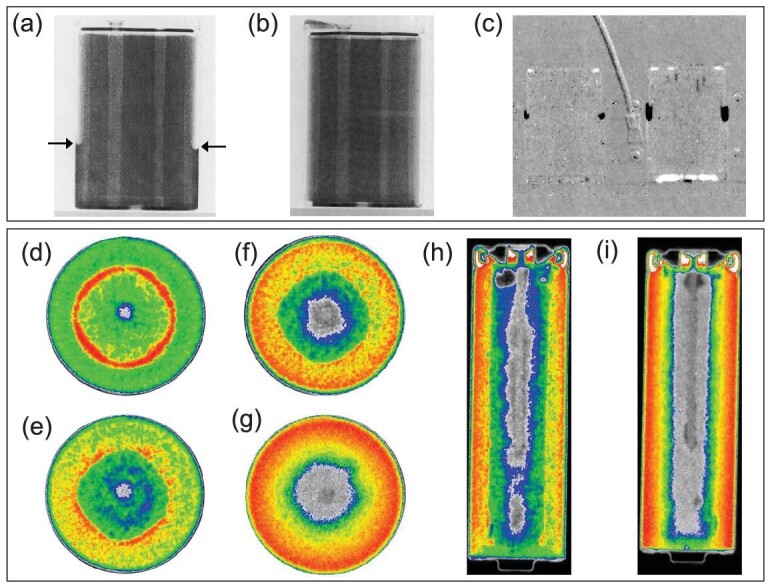
Application of NI in observing the electrolyte consumption in cells. (a) Neutron image of a fresh cell; the arrows point to the electrolyte level; (b) neutron image of a cell after 70 cycles; (c) referenced image of two cells placed side by side in the neutron beamline; the cell on the left had been charged to 380  mAh before the NI experiment, whereas the cell on the right was fresh at the beginning of the experiment. Reprinted with permission from ref [77]. Copyright 2001, Elsevier. (d) Axial slices through an alkaline cell in the fresh state; (e) axial slices through an alkaline cell after 1 Ah discharged at 1 A; (f and g) axial slices through the cell in the end life discharged at 1 A and at 0.05 A; (h and i) slices along the height of the cell in the end life discharged at 1 A and at 0.05 A. Reprinted with permission from ref [82]. Copyright 2010, Institute of Physics.

The electrolyte consumption in cells has a significant influence on the performance in terms of capacity retention and resistance, and the wettability inside the battery also affects the current density of local regions [88–90]. Lanz *et al.* [77] were the first to apply NI to reveal the consumption of excess electrolytes of commercial LIBs (type ICP-340848) after electrochemical cycling. As illustrated in Fig. 7(a), at the lower left and right edges of the casing (as indicated by the marked arrows), the excess liquid electrolyte in the fresh cell can be clearly visualized by NI due to the strong neutron beam attenuation of H and Li elements. By contrast, no excess electrolyte remained at the edges of the casing after 70 cycles (Fig. 7(b)), which revealed that the electrolyte was irreversibly consumed during charge and discharge. In addition, the changes in electrolyte distribution can be analyzed by constructing the ‘referenced images’. As presented in Fig. 7(c), the *in situ* visualization of the cells suggested displacements of excess electrolytes during initial charging, which may be attributed to the volume changes of the electrode and gas generation caused by SEI formation. Riley *et al.* [82] investigated the degree of anode utilization and changes in the electrolyte distribution of alkaline primary batteries by *in situ* neutron tomographic measurements. During discharge from the fresh state to 1 Ah (Fig. 7(d) and (e)), the ZnO product appeared near the Zn anode and spread towards the separator (red circled area), and the KOH electrolyte near the anode pin was consumed. On the other hand, they revealed that the degree of anode utilization at a low discharge current (0.05 A; Fig. 7(g) and (i)) was more complete than that at a high discharge current (1 A; Fig. 7(f) and (h)). The consumption of electrolyte is primarily associated with the reaction between it and the electrode materials during the charging and discharging of batteries. The combination of NI and electrochemical characterization can provide a more comprehensive analysis of the relationship between electrolyte consumption and battery performance.

## GASEOUS PHASE

Gas generation is generally caused by the electrolyte decomposition and structural release from cathode materials during the lifespan of batteries, which can seriously impact the performance of batteries, e.g. reduced lifetime, electrolyte displacement and increased impedance [91–94]. Besides, abuse conditions (e.g. overcharging, overheating) make the gassing worse or even lead to severe accidents [95–97]. Typically, gas generation does not occur during the low-voltage stage of the battery charging process. However, gas generation can occur due to electrolyte decomposition or the structural release from the cathode in the high-voltage stage.

**Figure 8.**
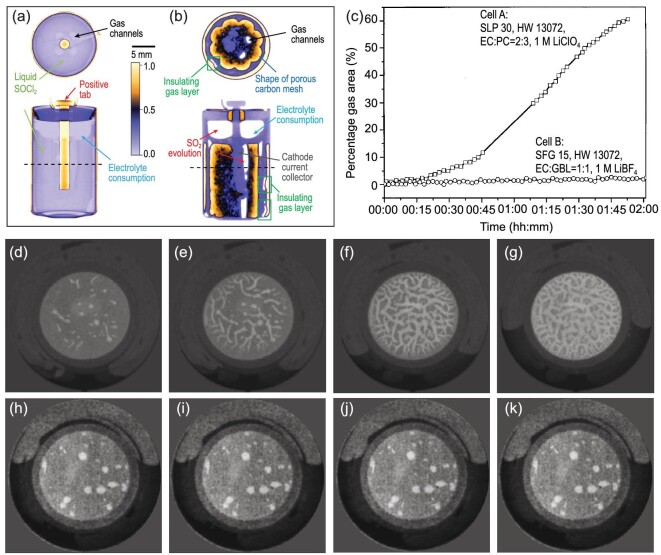
Application of NI in observing the gas generation in cells. (a and b) Horizontal and vertical orthogonal slices from the X-ray CT and neutron CT of the fully discharged Li/SOCl_2_ cells at 25 mA. Reprinted with permission from ref [59]. Copyright 2020, Institute of Physics. (c) Evolution of the percentage of the electrode area covered with gas bubbles for a propylene carbonate– (PC) based cell and a γ-butyrolactone- (GBL) based cell; (d–g) neutron images of gas evolution for a PC-based cell at different SoCs; (h–k) neutron images of gas evolution for a GBL-based cell at different SoCs. Reprinted with permission from ref [29]. Copyright 2004, Elsevier.

However, it is difficult to observe the evolution of gas through conventional imaging technology, due to the low contrast of the electron beam or X-ray (see Fig. 8(a)) to different components inside cells [9]. In contrast, as Fig. 8(b) indicates, the evolution of SO_2_ gas in a Li/SOCl_2_ cell can be distinctly observed by neutron tomography [59], which exhibits distinct advantages over X-ray CT (Fig. 8(a)). Therefore, as a supplementary probe, the neutron beam can be used to analyze the gas generation behavior in batteries.

The volume of gas production is strongly affected by the type of electrolyte in LIBs [98–100]. Goers *et al.* [29] demonstrated that NI is a powerful tool to investigate *in situ* the gas evolution on graphite electrodes in LIBs containing different polyvinylidene fluoride based gel-type electrolytes. As shown in Fig. 8(d)– (g), during the first charge step of the LIB assembled with the electrolyte containing propylene carbonate (PC), the volume of gas generation started to increase along various channels, particularly toward the top of the battery. With the assistance of differential electrochemical mass spectrometry [101], they concluded that gas (mainly composed of propylene) was formed during the reduction of PC on the graphite electrode surface, which corresponded to the irreversible electrolyte consumption. In contrast, a minor increase in the gas bubbles appeared in the electrolyte containing γ-butyrolactone (GBL), which exhibited stable electrochemical behavior (Fig. 8(h)–(k)). In addition, the percentage of electrode area covered by the generated gas can be quantitatively analyzed according to the neutron images (Fig. 8(c)), which offered the possibility to compare different electrolyte systems related to the gas generation rate.

**Figure 9.**
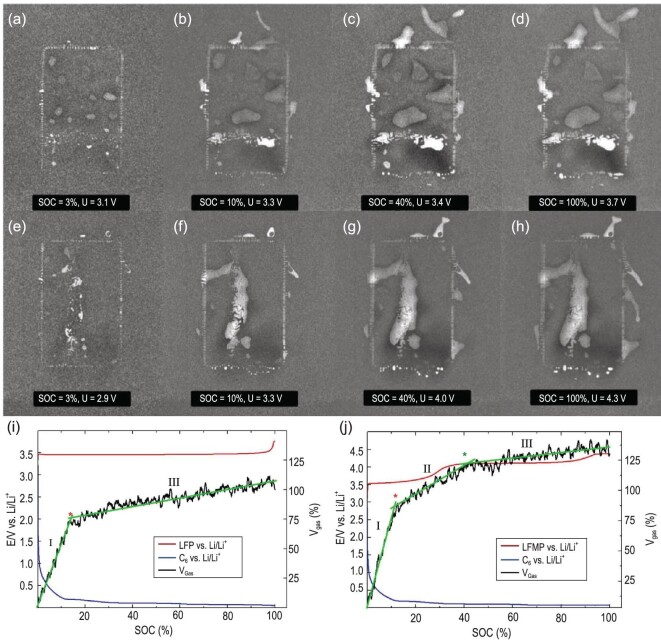
Neutron images of gas evolution in the LFP/graphite cell and LFMP/graphite cell at different SoCs. (a–d) Neutron images of gas evolution in the LFP/graphite cell at different SoCa; (e–h) neutron images of gas evolution in the LFMP/graphite cell at different SoCs; (i and j) gas volume calculated according to the NI data, and the changes in electrode potentials for LFP/graphite and LFMP/graphite cells at different SoCs. Reprinted with permission from ref [106]. Copyright 2017, Institute of Physics.

Also, the gas generation behavior in LIBs depends on the electrochemical and thermodynamic stability of the cathode materials [102–105]. Starke *et al.* [106], employing NI, studied the different characteristics of gas production caused by the presence of Mn in LiFe$\rm _x$Mn$\rm _{1-x}$PO_4_ (LFMP) in comparison to LiFePO_4_ (LFP). As presented in Fig. 9, the evolved gas was displayed in the form of bright areas, where the liquid electrolyte had been displaced due to the electrochemical processes at different SoCs. For both the LFP/graphite cell (Fig. 9(a)– (d)) and LFMP/graphite cell (Fig. 9(e)– (h)), the volume of gas production increased obviously from SoC = 3% to SoC= 10%, and remained nearly constant from SoC= 40% to SoC= 100%. However, according to the gas volume calculated on the basis of NI data (Fig. 9(i) and (j)), they found that approximately 30% more gas in the LFMP/graphite cell was generated in comparison to LFP/graphite. By combining the electrode potentials with the gas volume curves, they concluded that the continuously elevated gas evolution rate in section II of the LFMP/graphite cell (Fig. 9(j)) can be attributed to Mn insertion into the SEI layer on the anode.

Typically, the gas production process of batteries is intricate, influenced by various factors including electrolyte consumption, battery abuse, impurity introduction, inadequate packaging, etc. *In situ* monitoring of the gas distribution within the battery using NI, coupled with gas chromatography-mass spectrometry analysis to determine the gas composition, can provide valuable insights into the underlying chemical and electrochemical processes involved in gas generation.

## CURRENT DEVELOPMENT OF NEUTRON IMAGING

The development of neutrons as a tool for research has been driven by advances in instrumentation and experimental techniques. In recent years, neutron imaging has emerged as a powerful alternative to X-ray imaging for studying the internal structure and morphology features of materials. New neutron imaging modalities, such as phase contrast imaging [107], Bragg edge imaging [108] and polarized neutron imaging [109], provide more detailed information about the crystalline structure, magnetic domain walls and crystal orientation of materials [110]. As a result, neutron imaging is now being used by scientific researchers and even industrial users, expanding the scope and availability of these instruments for the benefit of the wider community. Besides, dual-mode tomography using neutrons and X-rays offers the potential for improved estimation of the composition of lithium batteries from the complementary interaction of the two probes with the materials [111].

(1) *Neutron source.* Research reactors are the main source of neutron imaging, but they are not portable and are only available at a few facilities [112]. The high cost of building, maintaining and the disposing of nuclear waste, along with safety and regulatory concerns, has led to the development of other sources of neutron production that can be used in the industry or for field applications. These sources include radioisotope-based neutron sources and accelerator-based neutron sources, offering reduced costs and increased mobility at the expense of lower neutron flux and poorer image quality [113]. As a result, these sources are only used in specific applications where portability, cost or on-field use is more important than high image quality. Meanwhile, spallation-based neutron sources are being developed in some research institutions to meet the higher requirements of scientific research [114]. Supplementary Table S2 lists some of the major international neutron sources across the globe that can be used for neutron imaging measurement for reference [115].

(2) *Advanced neutron imaging.* In traditional neutron imaging techniques, the intensity of the transmitted beam is modulated by inhomogeneous neutron attenuation, known as absorption contrast. However, this approach does not work with materials of high transmittance or when one is interested in probing the magnetic or electric field distribution inside a bulk. The concept of wave-particle duality of neutrons can be used to overcome these limitations and treat neutron-matter interaction on par with wave-matter interaction. This allows the concept of ‘phase of wave’ to be implemented in phase-based neutron imaging to improve existing techniques [110], such as Bragg edge imaging, neutron interferometry, analyzer-based imaging and free-space propagation-based neutron imaging. The potential for using diffraction contrast measurements with high spatial resolution has led to significant changes in the instrumentation at leading neutron imaging facilities around the world, as well as the development of new time-of-flight imaging instruments at powerful pulsed spallation sources worldwide [116–118]. Besides, the use of neutron spin-dependent interaction with matter allows for the mapping of electric and magnetic fields within the bulk of materials. Because of the high penetration depth of neutrons in most materials, polarized neutron imaging is uniquely effective, and it is even possible to combine phase-sensitive interaction with polarized neutrons to obtain anisotropic distributions in magnetic fields [119].

(3) *The neutron and X-ray tomography system.* Neutron tomography and X-ray tomography are both nondestructive, penetrating methods used to examine the internal structure and composition of materials. However, each method has its strengths and limitations, and using them separately can lead to incomplete analyses. In the past, researchers have tried to overcome this issue by using separate experimental setups for neutron and X-ray imaging [56,58,59], which is difficult to calibrate and prone to measurement errors. It also does not allow for real-time evaluation of the samples being measured, and different experimental conditions may impact the imaging results. To take full advantage of the complementary nature of neutrons and X-rays, researchers have recently developed neutron and X-ray tomography (NeXT) systems for incorporating both neutron and X-ray modalities to bring a full play to their respective advantages [111,120–123]. Factors such as spatial resolution, the field of view, dynamic range, acquisition time, proper sample positioning and accommodation of beam geometries are considered in the design of the NeXT system. Also, this combined neutron and X-ray imaging technology has potential applications in the evaluation of lithium batteries [121]. For example, neutrons can easily detect liquid electrolytes, the polymer separator, the Li-metal anode and the lithiation of graphite. Meanwhile, X-rays can distinguish the change in the cathode active material and physical damage, such as cracks, deformations and stresses.

(4) *The limitations of neutron imaging and strategies to enhance its functionality.* Currently, whether for a reactor or spallation-based neutron source, the flux, monochromaticity and divergence of the generated neutron beam are inferior to those of X-rays. Furthermore, the spatial resolution of detectors used for NI typically ranges in the order of tens of micrometers, which presents a notable limitation compared to the submicron-level imaging resolution achievable with X-rays. Therefore, the relatively lower measurement accuracy and imaging quality of NI hinder the advancement of high-resolution imaging techniques. Here are several strategies that can enhance the functionality of NI techniques in lithium batteries. Firstly, implementing Bragg-edge NI based on time-of-flight methods can be advantageous for characterizing lithium batteries. This approach allows for the acquisition of both morphology and structure information of the materials inside the batteries (e.g. the phase transition of cathode). Secondly, coupling the NI facility with X-ray imaging can provide complementary benefits. By combining these two imaging techniques, researchers can obtain a more comprehensive understanding of the evolution inside the batteries. Furthermore, the design of an *in situ* imaging device, specifically tailored for lithium batteries and located at the sample position of the NI facilities, can serve a highly functional purpose by enabling real-time observation of the internal evolution and dynamic processes within the batteries.

## SUMMARY AND PROSPECT

To summarize, we have reviewed the research progress of NI in observing lithium batteries based on the particularity of the interaction between neutron beams and matter. In terms of the solid phase, NI allows for the quantitative calibration of the lithium concentration distribution within the electrode, enabling *in situ* analysis of the lithiation/delithiation process. In addition, the dynamic evolution process of lithium dendrites can be visualized in the neutron images. Generally, the displacement of liquid electrolytes inside the batteries is coupled with gas generation. NI can be applied not only to observe the soaking process and consumption of electrolytes, but also to track the evolution of gas production in the battery.

**Figure 10.**
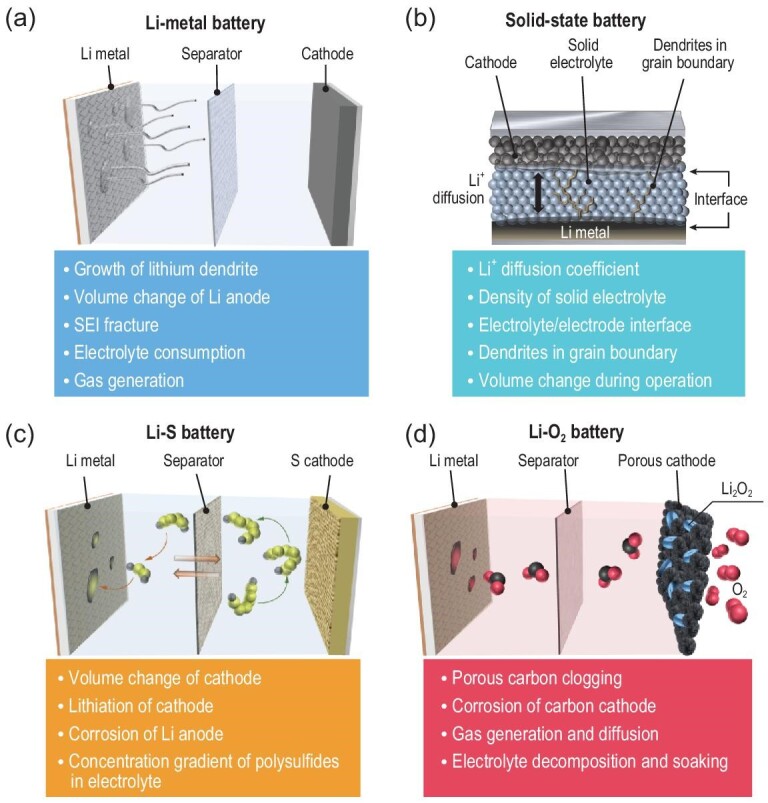
Future directions for the application of neutron imaging in advanced batteries. (a) Li-metal battery, (b) solid-state battery, (c) Li-S battery, (d) Li-O_2_ battery.

Despite being regarded as a potent tool for observing various states of matter within lithium batteries, the widespread use of NI has been hindered by the limited availability and high cost of neutron sources, particularly when compared to X-ray or electron beam sources. Consequently, NI has only found limited application in studies on traditional lithium batteries, and its usage in newly emerging cutting-edge batteries, such as Li-metal batteries, solid-state batteries, Li-S batteries and Li-O_2_ batteries, remains rare currently. Therefore, it is necessary to extend the powerful capabilities of NI to the characterization of advanced batteries. In light of this, we would like to propose several prospects for the future application of NI, specifically in observing the internal evolution of advanced batteries (Fig. 10).

1. *Li-metal batteries.* The lithium-metal anode possesses incomparable advantages in energy density, but there are still some formidable challenges such as the growth of lithium dendrite, volume change, SEI fracture and electrolyte consumption [124]. Taking advantage of the sensitivity of the neutron beam to Li metal, some specific *in situ* experiments can be designed to observe the evolution process of lithium dendrite and the volume in larger size lithium-metal batteries (e.g. pouch cell), to compensate for the shortage of *in situ* electron microscopy that is mainly applicable to smaller size cells. In addition, taking advantage of NI in liquid electrolytes and gas, the consumption of electrolytes and generation of gas caused by SEI fracture can be analyzed *in situ* (Fig. 10(a)). On the other hand, the traditional NI currently possesses relatively low spatial resolution of the order of tens of micrometers, resulting in the limitations in distinguishing features like lithium dendrites and SEI layers at the micron or submicron level. Hence, electron microscopy (e.g. SEM, TEM) is suggested to be employed to observe samples acquired through disassembling the battery, allowing for more localized morphology information in Li-metal batteries to compensate for the spatial resolution shortcomings of NI.
2. *Solid-state batteries.* Solid-state batteries have been considered to be the most promising next-generation batteries as a result of their great potential for improving safety and energy density benefits [125]. The mechanisms of ion diffusion and interface evolution involved in solid-state batteries can be investigated using NI (Fig. 10(b)). Firstly, the neutron absorption cross sections of Li^6^ and Li^7^ isotopes are very different. This natural property can be used to quantitatively calculate the lithium-ion diffusion coefficient of various solid electrolytes (e.g. oxides, sulfides and halides), as a supplement to the measurement of ionic conductivity by electrochemical impedance spectroscopy. Secondly, the density or porosity of the solid-state electrolytes, as well as the evolution of the interface morphology during the cycle of the solid-state batteries, can be visualized by neutron tomography. Thirdly, the gradient distribution of the lithium-ion concentration at various interfaces inside the solid-state batteries can be calibrated by NI, which can provide important information for the ion diffusion mechanism at the interfaces. Fourthly, NI can be applied to observe the dynamic evolution of lithium dendrite growth along the grain boundary of solid electrolytes *in situ*. Besides, the molds utilized during the assembly of solid-state batteries to ensure tight electrode-electrolyte contact can introduce interference with the transmitted neutron beam, potentially compromising image quality. To mitigate the adverse effects on the neutron beam and signals, molds for solid-state battery assembly can be designed and fabricated using materials with low neutron absorption (e.g. zirconium alloy).
3. *Li-S batteries.* Li-S batteries possess the merits of high energy density and low cost, making them promising candidates for rechargeable batteries in the future [126]. In addition to analyzing the gas production and dendrite growth mechanism similar to Li-metal batteries, the volume change in the sulfur cathode and shuttle effect in Li-S batteries can be studied through NI (Fig. 10(c)). During the charging and discharging process, the solid-liquid-solid transition of the cathode material (i.e. the conversion between S and Li_2_S) will cause an 80% volume change, which is associated with the lithiation of the cathode in Li-S batteries. Therefore, the evolution of cathode morphology can be observed *in situ* from both the volume change and lithium concentration through NI. On the other hand, the high-order polysulfide (Li_2_S$\rm _x$) dissolved in electrolyte tends to diffuse to the Li-metal anode due to the concentration gradient and cause irreversible reactions, thus causing severe anode corrosion of Li-S batteries. Also, the concentration gradient of polysulfides in the electrolyte implies that the lithium concentration is distributed differently, which is suitable for establishing quantitative correlation through neutron images. Still, the traditional NI technique faces challenges in distinguishing the various phases associated with polysulfide, resulting in the omission of crucial structural details during charging and discharging. To overcome this limitation, the Bragg-edge NI technique can be utilized to acquire phase information of polysulfide while simultaneously capturing morphology information. This integration allows for a more comprehensive understanding of the mechanisms underlying the shuttle effect of Li-S batteries, providing valuable insights into their performance and behavior.
4. *Li-O_2_ batteries.* Different from conventional lithium batteries, Li-O_2_ batteries are based on the reversible formation/decomposition of Li_2_O_2_ on the porous cathode material, which can deliver a large theoretical energy density of 3600 Wh/kg [127]. The Li-O_2_ battery system involves solid, liquid and gas components, thereby NI can fully play a role in the analysis of the internal morphology evolution (Fig. 10(d)). In addition to observing the evolution of the Li metal anode in Li-O_2_ batteries, similar to what is observed in Li-metal batteries, NI offers the capability to directly visualize the solid-phase-related problems of corrosion and pore-clogging in the carbon cathode. Meanwhile, the coupling problems of liquid and gaseous phases (i.e. electrolyte decomposition and soaking, gas generation and diffusion) can be observed in neutron images. Nonetheless, the data acquisition process of NI is often slower compared to X-ray imaging, resulting in limited temporal resolution. Consequently, this may lead to the loss of valuable information concerning complex thermodynamic and dynamic processes (e.g. reaction pathways, reaction sites, intermediate products). To obtain more comprehensive chemical/electrochemical information, it is advisable to complement NI with *in situ* spectroscopic characterization techniques, allowing for a deeper understanding of the intricate processes occurring within Li-O_2_ batteries.

Overall, lithium batteries heretofore have experienced remarkable leaps in the past decades with commercialization. Meanwhile, researchers are unremittingly developing advanced batteries aiming at expanding the boundaries of energy density, safety and cycle life. Still, these advanced batteries involve complex internal components, including solid, liquid and gaseous phases, as well as light elements (e.g. H, Li, O) widely existing in active materials, electrolytes and lithium metal. Therefore, we believe that NI can give full scope to its unique functions and advantages in the flourishing progress of lithium batteries in the future.

## Supplementary Material

nwad238_Supplemental_File
