# Supplementary material for: Application of neutron imaging in observing various states of matter inside lithium batteries
Source: Natl Sci Rev. 2023 Sep 15;10(11):nwad238. doi: 10.1093/nsr/nwad238 (PMC10581545; doi:10.1093/nsr/nwad238)
Supplement: nwad238_Supplemental_File [file nwad238_supplemental_file.pdf]

Application of neutron imaging in observing various states of matter inside lithium batteries

Table S1. Neutron scattering lengths and cross sections of common elements in lithium batteries[1].

Abbreviation	Unit	Quantity
E	--	element/isotope
Coh b	fm	coherent scattering length
Coh xs	barn	coherent scattering cross-section
Inc xs	barn	incoherent scattering cross-section
Scatt xs	barn	total scattering cross-section
Abs xs	barn	absorption cross section for thermal neutron (25 meV)

E	Coh b	Coh xs	Inc xs	Scatt xs	Abs xs
H	-3.7390	1.7568	80.26	82.02	0.3326
^{1}H	-3.7406	1.7583	80.27	82.03	0.3326
D(^{2}H)	6.671	5.592	2.05	7.64	0.000519
Li	-1.90	0.454	0.92	1.37	70.5
^{6}Li	2.00	0.51	0.46	0.97	940
^{7}Li	-2.22	0.619	0.78	1.4	0.0454
C	6.6460	5.551	0.001	5.551	0.0035
O	5.803	4.232	0.0008	4.232	0.00019
F	5.654	4.017	0.0008	4.018	0.0096
Na	3.63	1.66	1.62	3.28	0.53
Al	3.449	1.495	0.0082	1.503	0.231
Si	4.1491	2.163	0.004	2.167	0.171
P	5.13	3.307	0.005	3.312	0.172
S	2.847	1.0186	0.007	1.026	0.53
Cl	9.5770	11.5257	5.3	16.8	33.5
Ti	-3.438	1.485	2.87	4.35	6.09
V	-0.3824	0.0184	5.08	5.1	5.08
Mn	-3.73	1.75	0.4	2.15	13.3
Fe	9.45	1.22	0.4	11.62	2.56
Co	2.49	0.779	4.8	5.6	37.18
Ni	10.3	13.3	5.2	18.5	4.49
Cu	7.718	7.485	0.55	8.03	3.78
Se	7.970	7.98	0.32	8.3	11.7
Nb	7.054	6.253	0.0024	6.255	1.15
Ag	5.922	4.407	0.58	4.99	63.3
In	4.065	2.08	0.54	2.62	193.8
Sn	6.225	4.871	0.022	4.892	0.626

Table S2. Neutron imaging facilities of the major international neutron sources across the globe[2].

**Neutron source**	**Institute**	**Country**	**Beam intensity ($n{\cdot}cm^{-2}s^{-1}$)**	**Beam size (cm^{-2})**	**Applications/Functions**
**Reactor neutron sources**					
OPAL[3]	ANSTO	Australia	5.3×10^7 (L/D~500) 1.06×10^7 (L/D~1000)	20 × 20	Fuel cell, material science, palaeontology and cultural heritage
JRR-3M[4]	JAERI	Japan	1.5×10^8	Φ = 30.5 Φ = 25.5	Fuel cell, architecture, fluid
HANARO[5]	KAERI	Korea	2×10^7	35 × 45	Fuel cell, lithium battery, aircraft, heat exchanger
HFIR[6] (CG-1D)	ORNL	USA	2.2×10^7	8.6 × 8.6	Energy storage, biomedical, materials science, geosciences, cultural heritage
NCNR[7]	NIST	USA	2×10^8	10 × 10	Fuel cell, lithium battery, two-phase flow, porous media, heat pipes
HFR[8]	ILL	France	3×10^8	24 × 24	Physics, chemistry, biology, the production of radioisotopes for medical
FRM-II[9] (ANTARES)	MLZ	Germany	1×10^8	30 × 30	Standard neutron radiography, neutron CT, stroboscopic imaging, continuous radioscopy, phase contrast, energy/wavelength scan, polarized neutron imaging, grating interferometry
CARR[10]	CIAE	China	2×10^8 (L/D~293) 5×10^7 (L/D~585)	20 × 20	Lithium battery, fuel cell, aircraft, geosciences, cultural heritage, nuclear fuels
**Spallation neutron sources**					
JSNS-RADEN[11]	JPARC	Japan	9.8×10^7 (L/D~180) 5.8×10^7 (L/D~230)	30 × 30	Neutron radiography, neutron CT, Bragg-edge imaging, neutron resonance absorption imaging, polarized neutron imaging.

VENUS[12] (Under construction)	ORNL	USA	1×10^8 (white beam) 1×10^7 (TOF)	28 × 28	Bragg-edge imaging, texture mapping, grain, strain and porosity mapping in materials, energy storage materials, fracture propagation, magnetic properties
LANSCE-ERNI[13]	LANL	USA	1.5×10^7	28 × 28	Nuclear fuels and actinide materials, crystal growth, biology, fissile elements, energy-resolved neutron imaging
ISIS-IMAT[14]	RAL	UK	2×10^7	20 × 20	Aerospace and transportation, civil engineering, power generation, fuel cell, archaeology, earth and bioscience
ICON-NEUTRA[15]	PSI	Switzerland	1.3×10^7 (L/D~343) 3.9×10^6 (L/D~604)	15 × 15 30 × 30	Crystal growth, moisture content estimation and transport in building materials, dynamic imaging of two-phase flow, highly radioactive samples
CSNS-ERNI[16] (Under construction)	IHEP	China	9.8×10^7	30 × 30	Basic research, renewable energy materials, 3D distribution of microstructure in materials, engineering materials, cultural heritage and archaeological research, plant physiology, geology

REFERENCES

[1] Sears VF. Neutron scattering lengths and cross sections. Neutron news 1992; **3**: 26–37.

[2] Shukla M, Ray N and Patel T. Major neutron source facilities across the globe, 97–162, (Springer Singapore, Singapore2022).

[3] Garbe U, Randall T, Hughes C et al. A new neutron radiography/tomography/imaging station DINGO at OPAL. Physics Procedia 2015; **69**: 27–32.

[4] Matsubayashi M, Kobayashi H, Hibiki T et al. Design and characteristics of the JRR-3M thermal neutron radiography facility and its imaging systems. Nuclear technology 2000; **132**: 309–324.

[5] Wook Lee S, Joo Kim T, Soo Jeon J et al. Scientific review: neutron radiography at HANARO. Neutron News 2006; **17**: 13–19.

[6] Santodonato L, Bilheux H, Bailey B et al. The CG-1D neutron imaging beamline at the oak Ridge National Laboratory high flux isotope reactor. Physics Procedia 2015; **69**: 104–108.

[7] Arif M, Hussey DS, Baltic EM et al. Neutron imaging facility development and research trend at NIST. Physics Procedia 2015; **69**: 210–217.

[8] Arai M and Crawford K. Neutron sources and facilities, 13–30, (Springer US, Boston, MA2009).

[9] Tremsin AS, Dangendorf V, Tittelmeier K et al. Timeresolved neutron imaging at ANTARES cold neutron beamline. Journal of Instrumentation 2015; **10**: P07008.

[10] He LF, Han SB, Wei GH et al. Development and application of neutron imaging technique at China advanced research reactor. Materials Science Forum 2016; **850**: 153–160.

[11] Kiyanagi Y. Neutron imaging at compact acceleratordriven neutron sources in Japan. Journal of Imaging 2018; **4**: 55.

[12] Bilheux H, Herwig K, Keener S et al. Overview of the conceptual design of the future VENUS neutron imaging beam line at the Spallation Neutron Source. Physics Procedia 2015; **69**: 55–59.

[13] Borges NP, Losko AS and Vogel SC. Event centroiding applied to energy-resolved neutron imaging at LANSCE. Journal of Imaging 2018; **4**: 40.

[14] Kockelmann W, Zhang S, Kelleher J et al. IMAT–a new imaging and diffraction instrument at ISIS. Physics Procedia 2013; **43**: 100–110.

[15] Kaestner AP, Hartmann S, Kühne G et al. The ICON beamline – A facility for cold neutron imaging at SINQ. Nuclear Instruments and Methods in Physics Research Section A: Accelerators, Spectrometers, Detectors and Associated Equipment 2011; **659**: 387–393.

[16] Yang J, Zhou J, Jiang X et al. A novel energy resolved neutron imaging detector based on a time stamping optical camera for the CSNS. Nuclear Instruments and Methods in Physics Research Section A: Accelerators, Spectrometers, Detectors and Associated Equipment 2021; **1000**: 165222.
